# A novel machine-vision-based facility for the automatic evaluation of yield-related traits in rice

**DOI:** 10.1186/1746-4811-7-44

**Published:** 2011-12-12

**Authors:** Lingfeng Duan, Wanneng Yang, Chenglong Huang, Qian Liu

**Affiliations:** 1 Britton Chance Center for Biomedical Photonics, Wuhan National Laboratory for Optoelectronics-Huazhong University of Science and Technology, 1037 Luoyu Rd., Wuhan 430074, P.R. China; 2 Key Laboratory of Biomedical Photonics of Ministry of Education, College of Life Science and Technology, Huazhong University of Science and Technology, 1037 Luoyu Rd., Wuhan 430074, P.R. China

**Keywords:** Rice, Yield-related traits, Fast trait evaluation, Plant phenotyping, Machine vision

## Abstract

The evaluation of yield-related traits is an essential step in rice breeding, genetic research and functional genomics research. A new, automatic, and labor-free facility to automatically thresh rice panicles, evaluate rice yield traits, and subsequently pack filled spikelets is presented in this paper. Tests showed that the facility was capable of evaluating yield-related traits with a mean absolute percentage error of less than 5% and an efficiency of 1440 plants per continuous 24 *h *workday.

## Background

Rice is the staple food for a large number of countries and regions in the world, particularly in Asia [1]. Because the world's population is increasing, obtaining higher yields has been the primary breeding target of rice cultivation [2]. As a complex agronomic trait, rice yield is determined by the product of the grain weight, the number of grains per panicle and the number of panicles per plant. The number of total spikelets per panicle and the seed setting rate are two traits that multiplicatively determine the number of grains per panicle, and the grain weight is largely determined by the grain size, including the grain length, the grain width, and the grain thickness [3].

The evaluation of yield traits, including the number of total spikelets (including filled and unfilled spikelets), the number of grains (also known as the number of filled spikelets), the seed setting rate (the number of filled spikelets divided by the number of total spikelets), the 1000-grain weight, the grain length, and the grain width, is an essential step in rice breeding, genetic research and functional genomics research [4-6]. Currently, rice yield trait evaluation is mainly performed by experienced workers. When investigations of large numbers of plants are needed, the manual measurement process is very subjective, inefficient, tedious, and error-prone. Most importantly, manual measurements are greatly affected by worker fatigue, which is a major problem in conducting mass measurements and renders the evaluation results questionable. In addition to trait extraction and evaluation, data logging and seed management are two instrumental steps in rice research. Traditionally, the processing of data, seed packaging, and seed coding are preformed manually and are thus error-prone and unreliable. A mistake in data management and seed management would lead to incorrect decisions and treatment of the seeds and is thus intolerable in rice research. For this reason, at least three workers are normally needed to check and verify the data to avoid the mistakes.

Modern plant breeding technologies are able to produce hundreds to thousands of new varieties, creating the need for rapid evaluation of plant materials to provide pertinent information prior to entering the next cycle of selection [7]. The low efficiency of manual trait evaluation makes it unsuitable for meeting the increasing demand for higher evaluation speeds. Automated assessment and measurement of plant phenotypes is therefore indispensable [8]. Several efforts have been made to automate plant phenotyping, such as automated analysis of plant leaves [9], roots [10,11], hypocotyls [12], tillers [13], and shoot biomasses [14] and whole adult plants [15,16]. Plant phenotyping facilities have also been established in large research centers and universities in Australia [17,18], Germany [19], the UK [20], and France [21]. However, to the best of our knowledge, very little information is available on rice yield trait evaluation. Previously, our group developed a method to identify and count filled/unfilled spikelets [22]. We integrated visible-light imaging and X-ray imaging to simultaneously calculate the total spikelet number and the filled spikelet number. Nevertheless, the accuracy and efficiency of the method was limited by the performance of the X-ray system. Moreover, because of the utilization of an X-ray system, the prototype was expensive and posed a radiation hazard; thus, it was not suitable for widespread use. Research on single-trait evaluation, mainly grain dimension measurement, has also been reported [23,24]. However, these studies have the disadvantage of measuring only a single trait.

This work aimed to develop an integrated and labor-free engineering solution for automatic panicle threshing, rice yield-related trait evaluation (including the number of total spikelets (NTS), the number of filled spikelets (NFS), the 1000-grain weight (TGW), the grain length (GL), and the grain width (GW)), and seed packing. The task involved the development of an automated threshing machine for the threshing of spikelets and the separation of spikelets and other unwanted materials; the design of mechanisms for separating the filled spikelets from the unfilled spikelets; the design of a machine vision system for imaging rice spikelets; the development of real-time algorithms for trait evaluation; the construction of a data logging and management system for data tracking; and the design of control and communication procedures to supervise the whole system, including a user-friendly interface.

## Results and Discussion

### Development of the SEA facility

**Figure 1:**
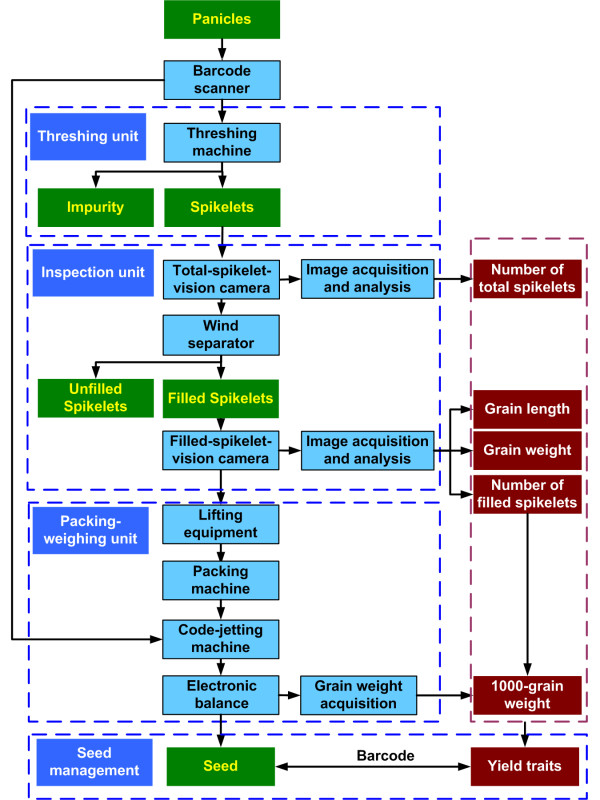
**Scheme of the SEA facility**. Panicles were threshed by the threshing unit, and spikelets were transferred to the inspection unit. A wind separator separated filled spikelets from unfilled spikelets. One camera acquired images of the total spikelets (including filled spikelets and unfilled spikelets) and one acquired images solely of the filled spikelets. The images were subsequently analyzed to obtain yield traits. After inspection, the filled spikelets were packed and weighed.

**Figure 2:**
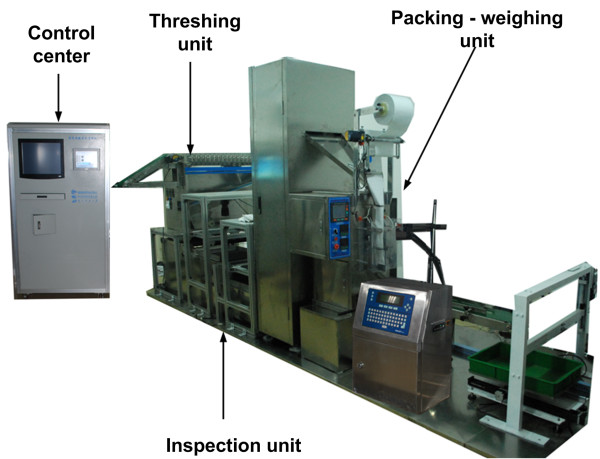
**The developed prototype of the SEA facility**. The facility mainly consisted of three units: a threshing unit for removing spikelets from the panicles, an inspection unit for assessing and measuring yield traits, and a packing-weighing unit for packing and weighing filled spikelets.

The facility consisted of three major elements: a threshing unit, an inspection unit, and a packing-weighing unit (Figure 1). The control center used software developed using LabVIEW 8.6 (National Instruments, USA) to control the whole system. The operating procedure includes the following steps: (1) the barcode of the rice plant being evaluated was obtained with a barcode scanner or by manual input, depending on the user's selection. (2) The threshing machine was started as panicles to be processed by the machine were detected. Spikelets were transferred to the inspection unit while impurities were collected at the impurity outlet. (3) The "total-spikelet-vision camera" collected images of the total spikelets (total-spikelet image) that came from the threshing unit (including filled and unfilled spikelets). A wind separator separated the filled spikelets from the unfilled spikelets. The 'filled-spikelet-vision camera' collected images of the filled spikelets (filled-spikelet image). The images were analyzed to determine yield traits. Unfilled spikelets were collected at the unfilled-spikelet outlet. (4) After inspection, lifting equipment raised the collected filled spikelets and delivered them to a packing machine for packaging. (5) A code-jetting machine printed the barcode of the recently examined rice plant on the packing bag. (6) An electronic balance weighed the filled spikelets and sent the data to the computer. (7) A collecting device collected the packed filled spikelets. An additional movie file shows the operation procedure in more detail [see Additional file 1].The developed prototype, dubbed the Seed-Evaluation Accelerator (SEA), is shown in Figure 2.

**Figure 3:**
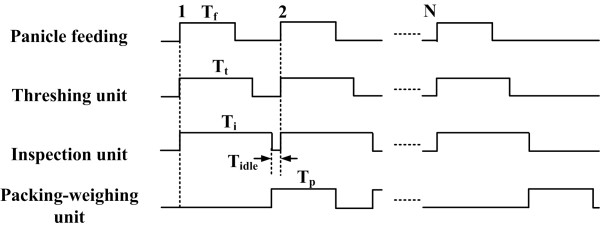
**Control flowchart of the instrument**.

The control flowchart is shown in Figure 3. The times required for panicle feeding (*T_f_)*, threshing (*T_t_*), inspection (*T_i_)*, and packing-weighing (*T_p_*) were designed to be 30, 50, 60, and 40 *seconds*, respectively. *T_idle _*was the idle time between subsequent measurements and depended on the operator. The threshing unit and the inspection unit worked in parallel with the packing-weighing unit, and T_p _was less than *T_i_*. Thus, the total processing time per rice plant was determined by Eq. 1:

(1) $$T=T_{i}+T_{idle}$$

#### Threshing unit

Panicles were fed into the threshing unit via a panicle inlet. The threshing of the spikelets was triggered by pulses received from a photoelectric sensor attached to the panicle inlet. The machine threshed the spikelets through roller-compaction processes. A sieve was used to separate the spikelets from branches and other unwanted materials. A tilted, vibrating plate under the sieve disaggregated the spikelets as they reached the end of the plate. After the spikelets were vibrated into the spikelet outlet, impurities were blown out through the impurity outlet by an air blower.

#### Inspection unit

**Figure 4:**
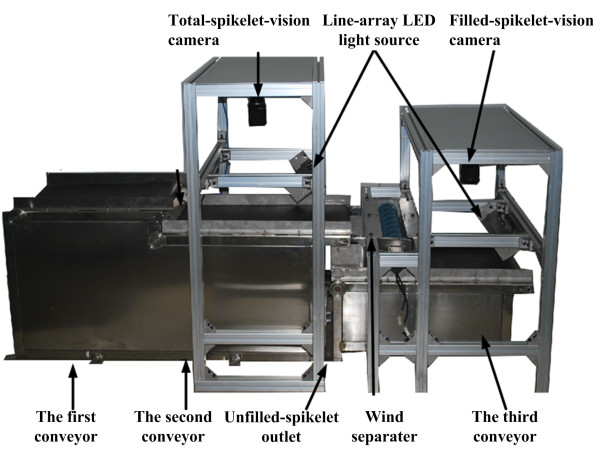
**Details of the inspection unit**. The inspection unit consisted mainly of a three-stage conveyor, two line-scan cameras, two LED light sources, a wind separator and an unfilled spikelet outlet.

Figure 4 shows the details of the inspection unit. The prototype used two line-scan cameras (Spyder 3 GIGE vision SG-11-02k80-00-R, Teledyne DALSA Company, Germany) to acquire 5000 × 2048 pixel grayscale images with a resolution of 0.23 *mm*/pixel. The cameras were controlled by the computer workstation (HP z600, Hewlett-Packard Development Company, USA) through an Ethernet card (NI PCIe 8235, National Instruments Corporation, USA) that digitized the images into 8-bit files. Two line-array LED light sources served as the illumination system.

Spikelets coming from the threshing unit were transferred onto the first conveyor (420 *mm *wide), where the ellipsoidal spikelets lay flat. When the spikelets were transferred to the second conveyor, they were imaged by the so-called "total-spikelet-vision camera" as they passed through the field of view of the camera. Then, the filled spikelets and the unfilled spikelets were separated by a wind separator mounted between the second and third conveyors. Unfilled spikelets were blown out and collected at an unfilled-spikelet outlet, whereas filled spikelets fell onto the third conveyor and were imaged by the "filled-spikelet-vision camera". To spread out the spikelets, the second conveyor was designed with a higher speed than that of the first conveyor, and the speed of the third conveyor was higher than that of the second. This design produced a separation of individual spikelets and consequently facilitated image analysis. A black conveyor belt was chosen, as it generated good contrast between the belt and spikelets and thus was beneficial for image segmentation.

#### Packing-weighing unit

**Figure 5:**
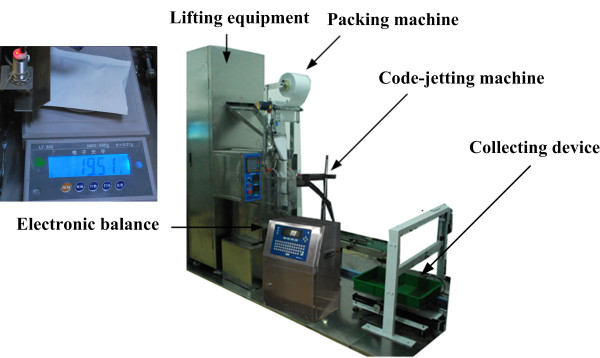
**Details of the packing-weighing unit**. The packing-weighing unit consisted of lifting equipment, a packing machine, a code-jetting machine, an electronic balance and a collecting device.

As shown in Figure 5, after inspection, the filled spikelets fell into a grain-collecting tank placed under the third conveyor. Next, lifting equipment raised the tank and poured the filled spikelets into a packing machine. After packing, a code-jetting machine printed the barcode of the plant being evaluated onto the packing bag. Subsequently, an electronic balance weighed the filled spikelets and sent the grain weight (*W_grain_*) to the computer. The TGW was obtained using Eq. 2:

(2) $$TGW=\left(W_{grain}\times 1000\right)/)NFS$$

#### Communication interface

**Figure 6:**
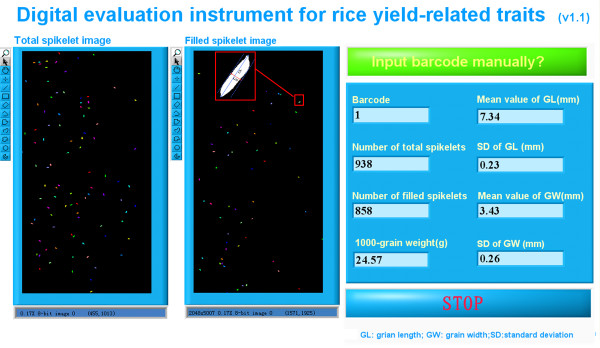
**Software interface of the implemented prototype**.

The communication interface is illustrated in Figure 6. The resultant total-spikelet image and the filled-spikelet image, along with the yield trait data, were displayed on the interface. When the "Input barcode manually?" button was clicked, a dialog box was shown to allow users to input the barcode manually.

#### Automated data and seed management

At the beginning of the yield trait evaluation for each rice plant, the user chose to either scan the barcode of the plant using a barcode scanner or input the barcode manually. This barcode was transferred to the code-jetting machine, which then sprayed the barcode on the packing bag to facilitate seed management. Yield trait data of the rice plant were stored in a Microsoft Excel file along with the barcode of the plant for indexing and data management.

### Performance evaluation of the hardware

Rice panicles from 214 harvested Huageng 295 rice plants were tested to evaluate the performance of the prototype. All of the spikelets, including spikelets at the impurity outlet, the unfilled-spikelet outlet and the filled-spikelet outlet (packing machine), were collected. The number of total spikelets and the number of filled spikelets at the three outlets were counted separately and recorded. For the NTS, NFS and TGW, each rice plant was evaluated three times by different personnel, and the average values were computed as reference data. Manual observations were defined and computed as given in Table 1.

**Table 1:** Definition and calculation of manual observations

Variable	Definition	Calculation
*NTS_io_*	Number of total spikelets at the impurity outlet	
*NTS_uo_*	Number of total spikelets at the unfilled-spikelet outlet	
*NTS_fo_*	Number of total spikelets at the filled-spikelet outlet (packing machine)	
*NFS_io_*	Number of filled spikelets at the impurity outlet	
*NFS_uo_*	Number of filled spikelets at the unfilled-spikelet outlet	
*NFS_fo_*	Number of filled spikelets at the filled-spikelet outlet (packing machine)	
*NTS_manual_*	Manually measured value of the number of total spikelets (*NTS *of a rice plant)	*NTS_manual _*= *NTS_io _*+ *NTS_uo _*+ *NTS_fo_*
*NFS_manual_*	Manually measured value of the number of filled spikelets (*NFS *of a rice plant)	*NFS_manual _*= *NFS_io _*+ *NFS_uo _*+ *NFS_fo_*
*NTS_manual.image_*	Manually measured value of the number of total spikelets that were imaged by the camera	*NTS_manual.image _*= *NTS_uo _*+ *NTS_fo_*
*NFS_manual.image_*	Manually measured value of the number of filled spikelets that were imaged by the camera	*NFS_manual.image _*= *NFS_uo _*+ *NFS_fo_*

**Figure 7:**
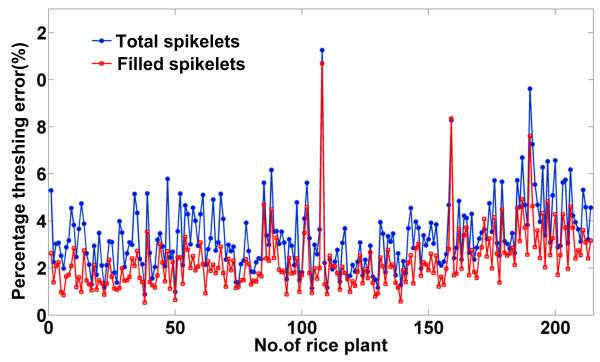
**Performance of the threshing unit**. The blue line and red line represent the percentage threshing errors of the threshing unit for total spikelets and filled spikelets, respectively.

The threshing machine worked well during the tests. The absolute threshing error for total/filled spikelets was calculated as the total/filled spikelet number at the impurity outlet. The percentage threshing error for total/filled spikelets was calculated as the absolute threshing error divided by the number of total/filled spikelets of the rice plant being evaluated. Figure 7 ill-ustrates the percentage error of the threshing unit for total spikelets and filled spikelets. As shown in Figure 7, the threshing error for total spikelets was higher than for filled spikelets, indicating that it was more difficult to thresh unfilled spikelets than filled spikelets. It can be observed from Figure 7 that some samples had threshing errors that were significantly higher than the average error. This was because the feeding speed had an important influence on the threshing of the spikelets. Two panicles entering the threshing machine at the same time would lead to fewer spikelets being threshed. These spikelets that remained on the panicles would be blown out with the panicle branches as impurities and, consequently, increase the threshing error. The mean absolute error and mean absolute percentage error of the threshing unit were 34 and 3.33%, respectively, for total spikelets and 19 and 2.27%, respectively, for filled spikelets.

The multi-stage conveyor proved able to segregate most of the spikelets. As a result, most kernels in both the total-spikelet image and the filled-spikelet image were observed to be isolated kernels. The packing-weighing unit worked properly during the test, with average weighing differences of less than 0.01 *g *between automatic measurements and manual measurements.

### Performance evaluation of the image analysis algorithms

**Figure 8:**
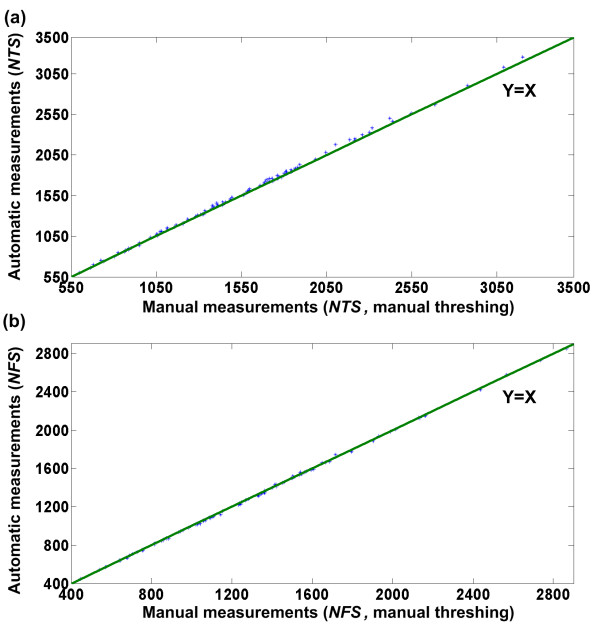
**Performance of the image analysis algorithms with manually threshed spikelets**. Scatter plots of manual measurements versus automatic measurements with manually threshed spikelets for the number of total spikelets and the number of filled spikelets are shown. Ninety Huageng 295 rice plants were used as samples in the evaluation experiment. Least squares linear regression produced the following results: (a) number of total spikelets: line of best fit: y = 1.01x-2.85, correlation coefficient r = 0.9995, (b) number of filled spikelets: line of best fit: y = 0.998x-1.65, correlation coefficient r = 0.9998.

**Figure 9:**
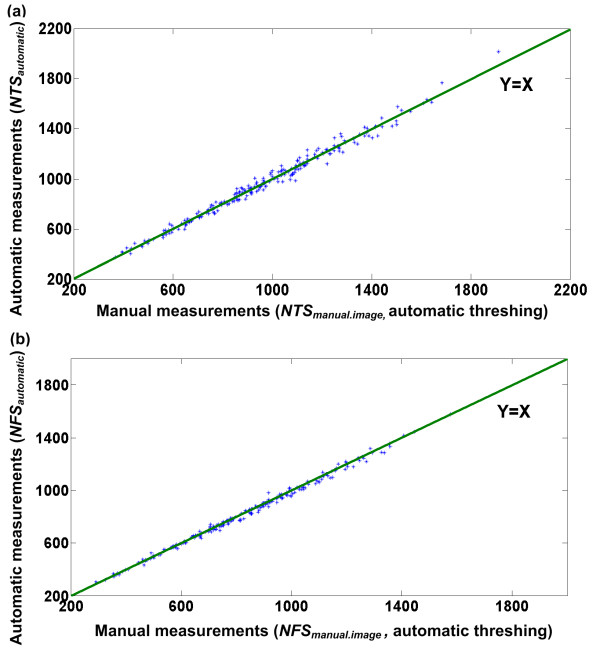
**Performance of the image analysis algorithms with automatically threshed spikelets**. Scatter plots of manual measurements versus automatic measurements with automatically threshed spikelets using the threshing unit for the number of total spikelets and the number of filled spikelets are shown. In total, 214 Huageng 295 rice plants were used as samples in the evaluation experiment. Least squares linear regression produced the following results: (a) number of total spikelets: line of best fit: y = 0.99x+6.07, correlation coefficient r = 0.993, (b) number of filled spikelets: line of best fit: y = 0.99x+4.47, correlation coefficient r = 0.997.

To test the performance of the image analysis algorithms, manually threshed spikelets of 90 Huageng 295 rice plants were fed into the inspection unit and imaged by the cameras. Manually threshed spikelets were used to exclude measuring errors caused by threshing. Figure 8 shows the results of manual observation versus image analysis of manually threshed spikelets. In comparison, the image-analysis performance with automatically threshed spikelets coming from the threshing unit was also investigated (Figure 9). The mean absolute error and the mean absolute percentage error with manually threshed spikelets were 22 and 1.36%, respectively, for the NTS and 7 and 0.54%, respectively, for the NFS. In comparison, the mean absolute error and the mean absolute percentage error with automatically threshed spikelets were 27 and 2.81%, respectively, for the NTS and 15 and 1.77%, respectively, for the NFS. As expected, the measuring accuracy for the manually threshed spikelets was higher than for the automatically threshed spikelets. This was because the threshing machine removed the hulls of some spikelets, and the broken hulls would appear in the image as impurities. The external appearance of some broken hulls was similar to a spikelet, and consequently, they would be mistakenly treated as spikelets. In addition, some brown rice spikelets (spikelets without hulls) were much smaller than normal spikelets and thus would be mistakenly treated as impurities.

As measuring the GL and the GW of a grain is troublesome to perform, the mean GL and GW of only 50 rice plants were measured manually. For each rice plant, 10 filled spikelets were randomly chosen. Five workers measured the grain length and the grain width of each spikelet using a Vernier caliper, and the average value was regarded as the grain length and the grain width of one spikelet. To eliminate measuring errors caused by sampling, the facility measured the same 10 spikelets of each rice plant that were used for the manual measurements. The GL and GW of each rice plant were computed as the mean grain length and grain width values of the selected 10 spikelets.

Large variances were noted for the GL and GW estimation among different personnel. The lack of a mathematical definition of GL and GW and worker fatigue under continuous measuring conditions were believed to be two primary reasons for the huge variance among workers. Manual measurements and automatic measurements for GL and GW are illustrated in Table 2. Generally, automatically measured GL values were slightly larger than the values from manual measurements. However, GW values that were measured automatically matched well with those measured manually. The larger GL value from automatic measurement than from manual measurements was because there were errors in the manual location of the maximum length (GL), and inaccurate location of the length invariably leads to underestimation of the GL. Unlike GL measurements, imprecise location of the width may result in underestimation or overestimation of the GW. As a result, the average manually measured GW values matched well with automatically measured values.

**Table 2:** Grain length and grain width measured manually versus automatically (sample size = 50)

**No**.	Grain length (mm)				Grain width (mm)			
	
	Automatic measurement	Manual measurement	Absolute error	Relative error	Automatic measurement	Manual measurement	Absolute error	Relative error
1	7.34	7.31	0.03	0.46%	3.43	3.37	0.06	1.82%
2	7.32	7.35	0.03	0.36%	3.31	3.41	0.10	2.85%
3	7.23	7.10	0.13	1.88%	3.33	3.21	0.12	3.85%
4	7.43	7.31	0.12	1.65%	3.43	3.27	0.16	4.91%
5	7.19	7.14	0.05	0.66%	3.19	3.23	0.04	1.25%
6	7.45	7.19	0.27	3.69%	3.29	3.23	0.06	1.91%
7	7.31	7.26	0.06	0.76%	3.19	3.24	0.05	1.54%
8	7.12	7.09	0.03	0.38%	3.22	3.32	0.10	2.94%
9	7.38	7.29	0.08	1.14%	3.32	3.29	0.03	0.84%
10	7.39	7.31	0.08	1.14%	3.30	3.33	0.03	0.81%
11	7.41	7.20	0.21	2.97%	3.24	3.28	0.04	1.23%
12	7.40	7.21	0.19	2.62%	3.29	3.22	0.07	2.15%
13	7.25	7.13	0.12	1.61%	3.29	3.33	0.04	1.11%
14	7.16	7.13	0.03	0.46%	3.29	3.23	0.07	2.07%
15	7.39	7.23	0.15	2.14%	3.26	3.32	0.06	1.86%
16	7.51	7.31	0.20	2.76%	3.28	3.33	0.05	1.39%
17	7.19	7.13	0.06	0.91%	3.26	3.28	0.02	0.52%
18	7.46	7.29	0.16	2.25%	3.37	3.40	0.03	0.79%
19	7.27	7.28	0.01	0.14%	3.30	3.30	0.00	0.02%
20	7.29	7.20	0.09	1.28%	3.32	3.36	0.04	1.33%
21	7.32	7.23	0.09	1.29%	3.24	3.33	0.09	2.57%
22	7.33	7.25	0.07	0.98%	3.29	3.36	0.07	2.15%
23	7.52	7.37	0.15	2.09%	3.24	3.39	0.15	4.48%
24	7.38	7.32	0.06	0.85%	3.28	3.37	0.08	2.48%
25	7.33	7.33	0.00	0.01%	3.32	3.43	0.11	3.07%
26	7.36	7.22	0.14	1.94%	3.29	3.31	0.02	0.65%
27	7.36	7.19	0.17	2.39%	3.33	3.29	0.04	1.09%
28	7.28	7.29	0.01	0.14%	3.31	3.29	0.02	0.67%
29	7.24	7.18	0.06	0.81%	3.27	3.29	0.02	0.60%
30	7.42	7.28	0.15	2.00%	3.48	3.41	0.07	2.05%
31	7.33	7.27	0.06	0.82%	3.24	3.33	0.09	2.71%
32	7.29	7.11	0.19	2.61%	3.36	3.27	0.10	2.94%
33	7.47	7.35	0.12	1.68%	3.31	3.31	0.00	0.03%
34	7.26	7.24	0.02	0.21%	3.41	3.41	0.00	0.00%
35	7.59	7.42	0.17	2.22%	3.42	3.39	0.03	0.92%
36	7.31	7.18	0.13	1.80%	3.26	3.21	0.05	1.41%
37	7.36	7.20	0.16	2.16%	3.35	3.33	0.02	0.47%
38	7.50	7.26	0.23	3.22%	3.34	3.30	0.05	1.42%
39	7.36	7.28	0.08	1.09%	3.36	3.35	0.01	0.28%
40	7.12	7.13	0.02	0.26%	3.14	3.17	0.03	0.80%
41	7.34	7.15	0.19	2.67%	3.32	3.31	0.01	0.24%
42	7.21	7.26	0.05	0.69%	3.24	3.31	0.08	2.29%
43	7.39	7.20	0.19	2.64%	3.36	3.38	0.02	0.46%
44	7.37	7.24	0.13	1.75%	3.35	3.37	0.02	0.48%
45	7.38	7.32	0.06	0.78%	3.32	3.38	0.06	1.74%
46	7.24	7.20	0.03	0.46%	3.37	3.35	0.02	0.59%
47	7.42	7.26	0.16	2.23%	3.30	3.34	0.05	1.41%
48	7.59	7.39	0.21	2.79%	3.35	3.36	0.01	0.21%
49	7.38	7.32	0.05	0.73%	3.37	3.39	0.02	0.63%
50	7.28	7.23	0.05	0.68%	3.30	3.30	0.00	0.10%

Mean value	7.34	7.24	0.11	1.47%	3.31	3.32	0.04	1.31%

### Performance evaluation of the whole facility

**Figure 10:**
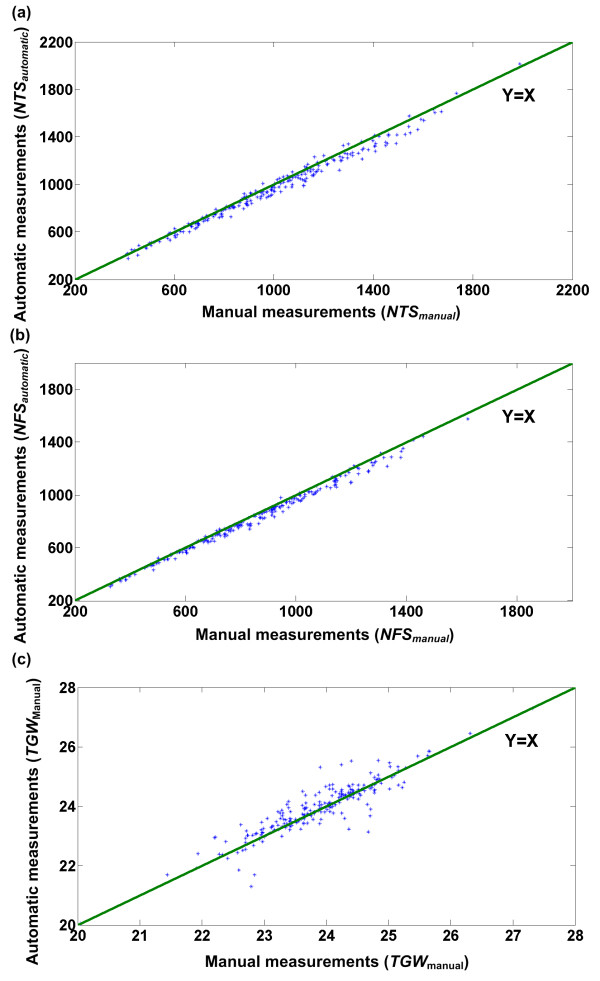
**Performance of the entire facility**. Scatter plots of manual measurements versus automatic measurements with the facility for (a) the number of total spikelets, (b) the number of filled spikelets, and (c) the 1000-grain weight are shown. In total, 214 Huageng 295 rice plants were used as samples in the evaluation experiment. Least squares linear regression produced the following results: (a) number of total spikelets: line of best fit: y = 0.96x+10.24, correlation coefficient r = 0.992, (b) number of filled spikelets: line of best fit: y = 0.96x+6.14, correlation coefficient r = 0.996, (c) 1000-grain weight: line of best fit: y = 0.94x+1.56, correlation coefficient r = 0.91.

Scatter plots of manual measurements versus automatic measurements of the whole facility for the number of total spikelets, the number of filled spikelets and the 1000-grain weight are shown in Figure 10. As weighing differences between automatic measurements and manual measurements were minor, the discrepancy in the TKW between manual and automatic measurements was chiefly caused by NFS measurement differences.

Table 3 summarizes the mean absolute error (MAE, defined by Eq. 3) and the mean absolute percentage error (MAPE, defined by Eq. 4) of the whole facility for the evaluated yield traits. As shown in the table, the facility was capable of evaluating yield traits with mean absolute percentage errors of 4.02%, 3.33%, 1.47%, 1.31% and 1.08% for number of total spikelets, number of filled spikelets, grain length, grain width, and 1000-grain weight, respectively. The MAE and MAPE were computed using Eqns. (3) and (4)

**Table 3:** Mean absolute error (MAE) and mean absolute percentage error (MAPE) for the evaluated traits (^a^sample size = 214, ^b^sample size = 50)

Trait	Definition	MAE	MAPE
NTS^a^	Number of total spikelets of a rice plant	41	4.02%
NFS^a^	Number of filled spikelets of a rice plant	29	3.33%
GL^b^	Average grain length of a rice plant	0.11 *mm*	1.47%
GW^b^	Average grain width of a rice plant	0.04 *mm*	1.31%
TGW^a^	1000-grain weight of a rice plant	0.26 *g*	1.08%

(3) $$\text{MAE}\;=\frac{1}{n}\overset{n}{\underset{i=1}{\sum }}|x_{i.a}-x_{i.m}|$$

(4) $$\text{MAPE}=\frac{1}{n}\overset{n}{\underset{i=1}{\sum }}\frac{|x_{i.a}-x_{i.m}|}{x_{i.m}}$$

where *n *was the number of samples, *x_i.a_*was the *i_th _*automatically measured value, and *x_i.m _*was the *i_th _*manually measured value.

As observed from the results, the measuring error of the NTS was larger than that of the NFS. This was because broken hulls caused by threshing were also imaged by the "total-spikelet-vision camera". Additionally, larger errors can result from broken hulls, which are very similar in appearance to complete spikelets and, consequently, may be treated as spikelets by the vision system but are ignored in manual observation. These broken hulls were blown out into the impurity outlet by the wind separator and thus would not influence the NFS measurement. Anther reason for the larger measuring error of the NTS was that the threshing error of the total spikelets was larger than that of the filled spikelets.

**Figure 11:**
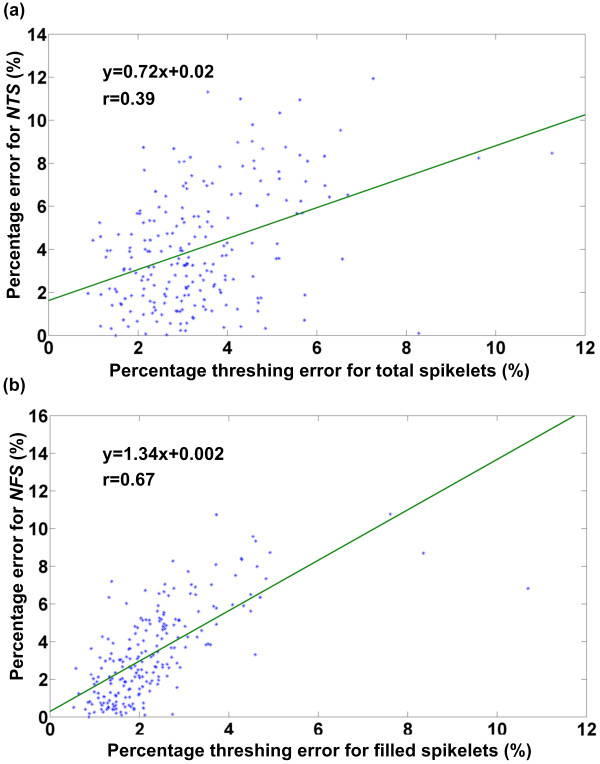
**Relationship between the percentage measuring error of the facility and the percentage threshing error**. (a) number of total spikelets and (b) number of filled spikelets.

The threshing error directly decreases the number of spikelets that pass through the inspection unit and, consequently, influences the measuring accuracy of the NTS and the NFS. The relation between the threshing error and the measuring error of the NTS and the NFS were investigated. Figure 11 shows the variation of the percentage measuring error of the facility for the NTS and the NFS as a percentage threshing error changes. As shown in Figure 11, the measuring error for both the NTS and the NFS presented an upward trend as the threshing error increased. Compared with the measuring error for the NFS, the measuring error for the NTS had a weaker relationship with the threshing error. This was because broken hulls had a considerable effect on the NTS measuring error.

### Measuring the efficiency of the whole facility

Assuming *T_idle _*was 0 s, the total processing time per rice plant was approximately 60 s (calculated as Eq. 1); i.e., the implemented prototype was able to perform yield trait evaluations for approximately 1440 rice plants per 24 h continuous workday. Generally, an experienced worker can evaluate approximately 20 plants per day (working 8 hours per day). As the prototype needs no human operation except for panicle feeding, it is feasible to run the facility continuously for 24 hours for mass measurements. From this point of view, the facility was capable of evaluating rice yield traits with an efficiency more than 70 times greater than that of manual operation at its maximum throughput.

The efficiency of the whole facility was determined by the inspection unit and the time elapsed, which was limited by the threshing time. More effective threshing methods will be developed in the future to improve the efficiency of the whole facility. The software was designed to allow the images to be processed concurrently as the cameras were acquiring images. Image processing requires less time than image acquisition. The shorter time needed for image processing also opened up the possibility of applying more sophisticated algorithms for more sophisticated solutions. For instance, statistical classifiers such as the distance classifier, discriminant analysis and artificial neural networks could be adapted in the future to discriminate broken hulls from spikelets.

### Automation and integration of threshing, multi-trait measurement and seed packing

With the only manual operation being panicle feeding, the SEA facility automated the entire process of threshing, fast multi-trait measurement and seed packing. The yield traits were automatically observed and stored with a unique code after system inspection. Meanwhile, the seeds were automatically packed with the relevant code. The automation and integration of the entire process will substantially improve the yield trait evaluation process for rice researchers. With the data tracking ability, it was convenient for the user to manage and analyze data. Data tracking also allowed the user to combine yield traits with other traits such as the tiller number, the leaf area, the plant height, thus allowing an integrated understanding. Moreover, data tracking is beneficial for seed management. Compared with manual data logging and seed management, the data tracking of the facility made data management and seed management more robust and reliable.

## Conclusions

This paper described an engineering prototype for the automatic evaluation of rice yield traits, including the number of total spikelets, the number of filled spikelets, the grain length, the grain width, and the 1000-grain weight. The prototype comprised three major units: the threshing unit, the inspection unit, and the packing-weighing unit. The mean absolute percentage error was less than 5% for all of the evaluated yield traits, and the efficiency was approximately 1440 plants per 24-hours continuous workday. The facility will be helpful for improving the accuracy and efficiency of rice yield trait evaluation and will serve as a powerful tool in rice plant phenotyping, which will eventually benefit rice breeding, genetic research, functional genomics research and other rice research. With some modifications, the application could be extended and generalized to other crops, such as wheat, corn and barley. Other compound yield traits such as the seed setting rate and the length-width ratio can also be deduced from the extracted traits. In summary, using agricultural photonics, the high-throughput facility, dubbed the Seed-Evaluation Accelerator, gives plant scientists a novel tool to unlock the phenotypic information coded in rice genome [25].

## Methods

### Image acquisition

**Figure 12:**
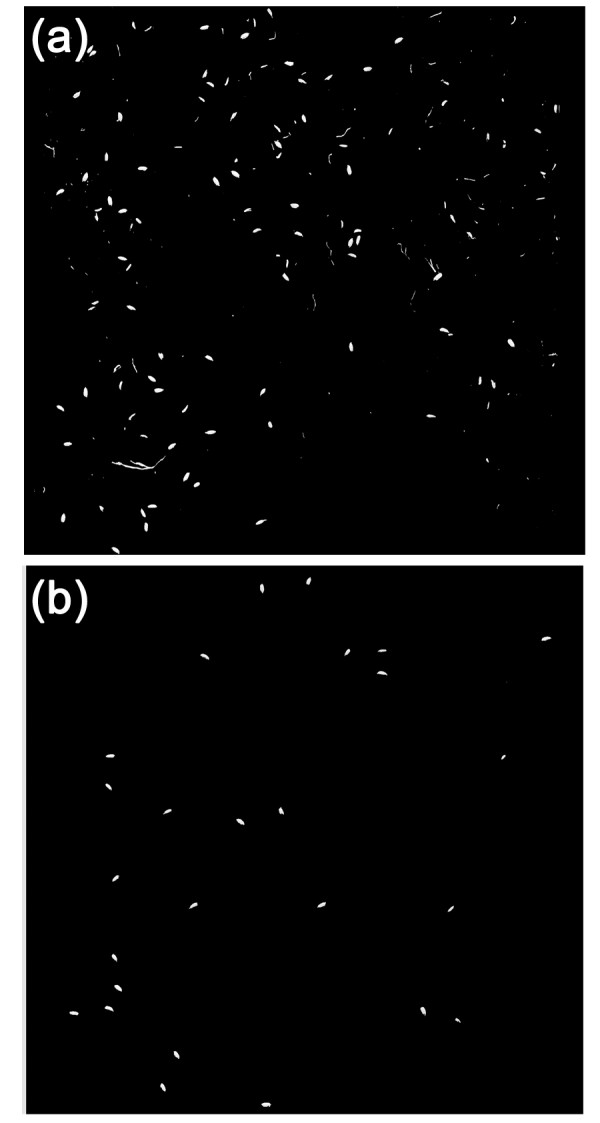
**Typical grayscale images for (a) a total-spikelet image and (b) a filled- spikelet image**.

The control software was designed for evaluating yield traits of one rice plant at a time. The continuous acquisition of the images was controlled by the pulses received from a photoelectric sensor attached to the panicle inlet of the threshing unit. Images were acquired and stored using the NI-IMAQ Virtual Instruments (VI) Library for LabVIEW (National Instruments Corporation, USA). For each plant, 14 images were acquired by the "total-spikelet-vision camera" (called the total-spikelet image) and 20 images were acquired by the "filled-spikelet-vision camera" (called the filled-spikelet image). This design was applicable to most rice varieties with less than 20 panicles per plant. Figure 12 shows typical total-spikelet and filled-spikelet images acquired by the two cameras. Note that the images shown in Figure 12 have been cropped for better visualization, as the original images are too large (5000 × 2048 pixels).

### Image analysis

The image-analysis software was programmed using NI Vision for LabVIEW 8.6 (National Instruments Corporation, USA). The software was designed to allow the two cameras (the "total-spikelet-vision camera" and the "filled-spikelet-vision camera") to work at the same time, and the images were analyzed in the computer simultaneously while the cameras were acquiring new images, thus optimizing the measurement efficiency.

**Figure 13:**
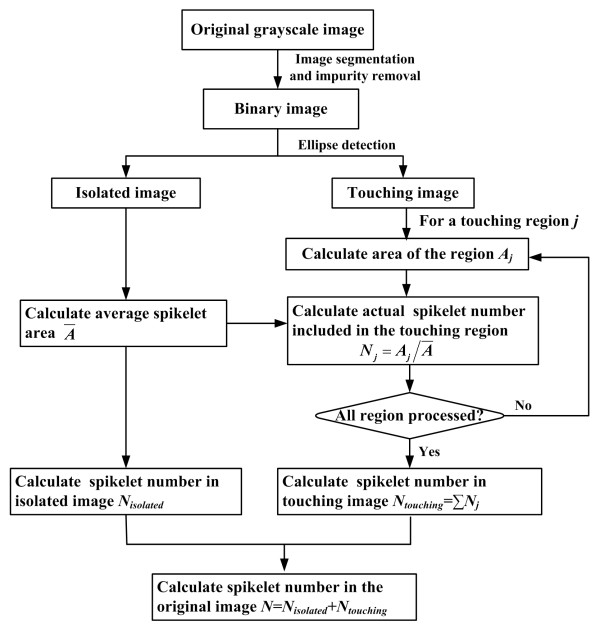
**Determination of spikelet number in an image**.

Figure 13 outlines the flowchart of the algorithm for determining the spikelet number in an image (a total-spikelet image or a filled-spikelet image). Image segmentation was performed to determine the background and objects of interest. For better processing speed, a pixel-oriented segmentation algorithm was applied in this study. A pixel was considered to belong to a background point if its grayscale fell below a pre-defined, fixed threshold. In a subsequent step, a median filter (3 × 3 neighborhood) was used to remove isolated pixels. As some small pieces of branches may appear on the conveyor, objects with a length-width ratio greater than three times that of the spikelets were treated as branches and removed. Branches with a length-width ratio less than three times that of the spikelets were subsequently removed using the "IMAQ detect line" operation. Small regions with an area less than half of the average area of spikelets were regarded as impurities and removed from the image. Spikelets may be touching during on-line processing. The shape of an isolated spikelet is roughly elliptical, so the "IMAQ detect Ellipse" operation was used to identify the isolated spikelet in the image (NI Vision Concepts Manual, National Instruments Corporation, USA). After identification, the original image was divided into two images, one image with only the isolated spikelet (isolated image) and the other with only the touching spikelets (touching image). From an efficiency perspective, we opted for a simple area-determination method to determine the spikelet number in the touching image *N_touching_*, which was computed by Eq. 5 and Eq. 6:

(5) $$N_{j}=\text{round}\;\left(A_{j}/)\bar{A}\right)$$

(6) $$N_{touching}=\overset{T}{\underset{j=1}{\sum }}N_{j}$$

where *N_j _*was the actual spikelet number for a given touching region *j *in the touching image, the function round(*x*) rounded *x *to the nearest integer, *A_j _*was the area of the touching region *j*, Ā was the average spikelet area calculated from the isolated image, and *T *was the number of touching regions.

The spikelet number (*N*) in the original image was determined by summing up the spikelet numbers in the isolated image (*N_isolated_*) and in the touching image (*N_touching_*). The NTS of the evaluated plant was calculated as the sum of the spikelet numbers in all 14 total-spikelet images. Similarly, the NFS of the evaluated plant was calculated as the sum of the spikelet numbers in all 20 filled-spikelet images.

The length and width of each isolated spikelet in the filled-spikelet images were calculated. The GL is defined as the maximum Euclidean distance between two boundary points of a filled spikelet, and the GW is defined as the maximum length of straight lines perpendicular to the line of the GL.

**Figure 14:**
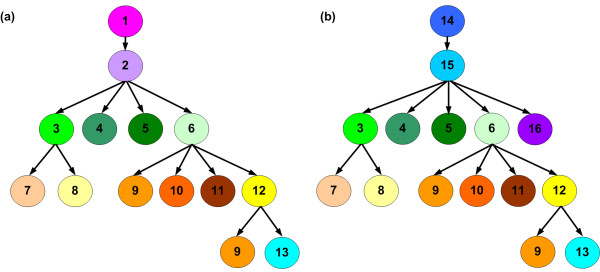
**Structure of image processing program in LabVIEW for (a) total-spikelet images and (b) filled-spikelet images**. 1: Source code file 1 (ImProcessPerPlant_TotalSpikeletImage.vi), 2: Source code file 2 (ImageAnalysis_TotalSpikeletImage.vi), 3: Source code file 3 (ImagePreProcess.vi), 4: Source code file 4 (Merge.vi), 5: Source code file 5(Split.vi), 6: Source code file 6 (ImageProcess.vi), 7: Source code file 7 (ImpurityRemove.vi), 8: Source code file 8 (StemRemove.vi), 9: Source code file 9 (MeanAreaCalculation.vi), 10: Source code file 10 (RemoveSmallParticle.vi), 11: Source code file 11 (GrainClassification.vi), 12: Source code file 12 (TotalGrainNumberCalculation.vi), 13: Source code file 13 (TouchingGrainNumberCalculation.vi), 14: Source code file 14 (ImProcessPerPlant_FilledSpikeletImage.vi), 15: Source code file 15 (ImageAnalysis_FilledSpikeletImage.vi), 16: Source code file 16 (GetLengthWidthRatio.vi). Illustrations showing source code files 1, 2, 3, 4, 5, 6, 7, 8, 9, 10, 11, 12, 13, 14, 15, and 16 are attached in Additional files 2, 3, 4, 5, 6, 7, 8, 9, 10, 11, 12, 13, 14, 15, 16, and 17, respectively.

Figure 14 shows the structure of the image-processing program in LabVIEW for total-spikelet images and filled-spikelet images. Illustrations showing block diagrams of the VIs developed in this research are attached in Additional files 2, 3, 4, 5, 6, 7, 8, 9, 10, 11, 12, 13, 14, 15, 16, and 17.

## Competing interests

The authors declare that they have no competing interests.

## Authors' contributions

LD developed the software, performed the evaluation experiment, analyzed the data, and drafted the manuscript. WY designed and built the hardware, performed the evaluation experiment, and contributed in writing the manuscript. CH provided support with hardware development and implementation of the evaluation experiment. QL supervised the study and contributed in writing the manuscript. All of the authors read and approved the final manuscript.

## Supplementary Material

Additional file 1 **Operating procedure of the facility**. A video showing the detailed operating procedure of the SEA facility. Click here for file

Additional file 2 **Source code file 1**. ImProcessPerPlant_TotalSpikeletImage.vi was used for processing total-spikelet images of one plant (14 images in the developed facility). The number of total spikelets of the evaluated plant was calculated as the sum of the spikelet numbers in all 14 total-spikelet images. Note that in the facility, ImProcessPerPlant_TotalSpikeletImage.vi functions were included in a 'queue' structure to allow images to be analyzed in the computer simultaneously while the cameras were acquiring new images, thus optimizing the measuring efficiency. Click here for file

Additional file 3 **Source code file 2**. ImageAnalysis_TotalSpikeletImage.vi was developed for processing a single total-spikelet image. Click here for file

Additional file 4 **Source code file 3**. ImagePreProcess.vi executed image segmentation and impurity removal. Click here for file

Additional file 5 **Source code file 4**. In continuous image acquisition, some grains may exist both in the bottom border of the previous image and in the top border of the subsequent image. Merge.vi. merged the object at the bottom border of the previous image with the other part of the object in the subsequent image. Click here for file

Additional file 6 **Source code file 5**. Split.vi extracted objects at the bottom border in the current image. Click here for file

Additional file 7 **Source code file 6**. ImageProcess.vi removed small particles and calculated spikelet number in an image. Click here for file

Additional file 8 **Source code file 7**. ImpurityRemove.vi removed objects with a length-width ratio greater than three times that of the spikelets. Click here for file

Additional file 9 **Source code file 8**. StemRemove.vi removed branches with length-width ratios less than three times that of the spikelet using the "IMAQ detect line" operation. Click here for file

Additional file 10 **Source code file 9**. MeanAreaCalculation.vi calculated the average area of the spikelets. Click here for file

Additional file 11 **Source code file 10**. RemoveSmallParticle.vi removed small regions with an area less than the defined area threshold. Click here for file

Additional file 12 **Source code file 11**. GrainClassification.vi divided the original image into two images: one image with only isolated spikelets (isolated image) and the other with only touching spikelets (touching image). Click here for file

Additional file 13 **Source code file 12**. TotalGrainNumberCalculation.vi determined the spikelet number in the original image by summing up the spikelet number in the isolated image and the spikelet number in the touching image. Click here for file

Additional file 14 **Source code file 13**. TouchingGrainNumberCalculation.vi calculated the spikelet number in the touching image. Click here for file

Additional file 15 **Source code file 14**. ImProcessPerPlant_FilledSpikeletImage.vi was used for processing filled-spikelet images of one plant (20 images in the developed facility). The number of filled spikelets of the evaluated plant was calculated as the sum of the spikelet numbers in all 20 filled-spikelet images. Note that in on-line measurements, ImProcessPerPlant_FilledSpikeletImage.vi functions were included in a 'queue' structure to allow images to be analyzed in the computer simultaneously while the cameras were acquiring new images. Click here for file

Additional file 16 **Source code file 15**. ImageAnalysis_FilledSpikeletImage.vi was developed for processing a single filled-spikelet image. Click here for file

Additional file 17 **Source code file 16**. GetLengthWidthRatio.vi calculated the length, width, and length-width ratio for each isolated grain. Click here for file
