# Supplementary figures and images for: A novel machine-vision-based facility for the automatic evaluation of yield-related traits in rice
Source: Plant Methods. 2011 Dec 12;7:44. doi: 10.1186/1746-4811-7-44 (PMC3264518; doi:10.1186/1746-4811-7-44)

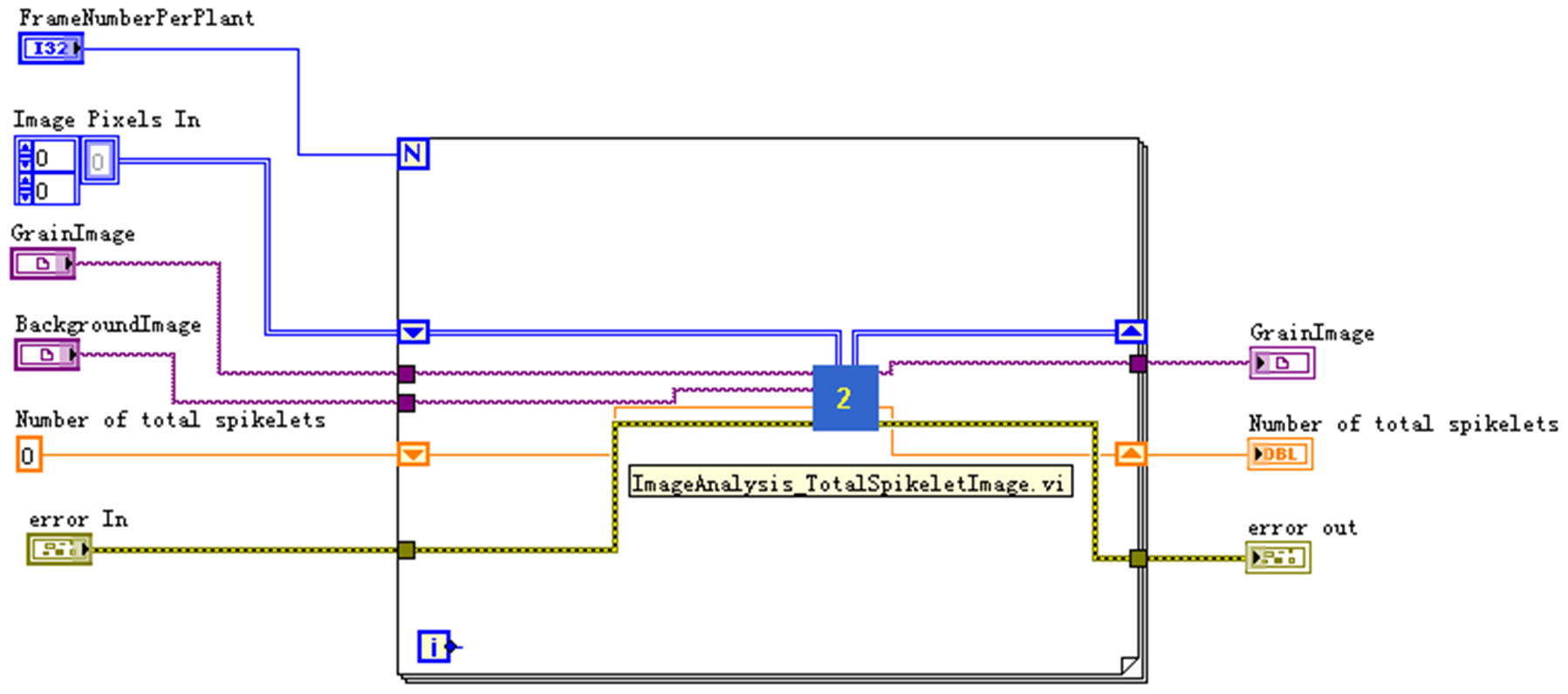

Supplement: Additional file 2 — Source code file 1. ImProcessPerPlant_TotalSpikeletImage.vi was used for processing total-spikelet images of one plant (14 images in the developed facility). The number of total spikelets of the evaluated plant was calculated as the sum of the spikelet numbers in all 14 total-spikelet images. Note that in the facility, ImProcessPerPlant_TotalSpikeletImage.vi functions were included in a 'queue' structure to allow images to be analyzed in the computer simultaneously while the cameras were acquiring new images, thus optimizing the measuring efficiency. [file 1746-4811-7-44-S2.TIFF]

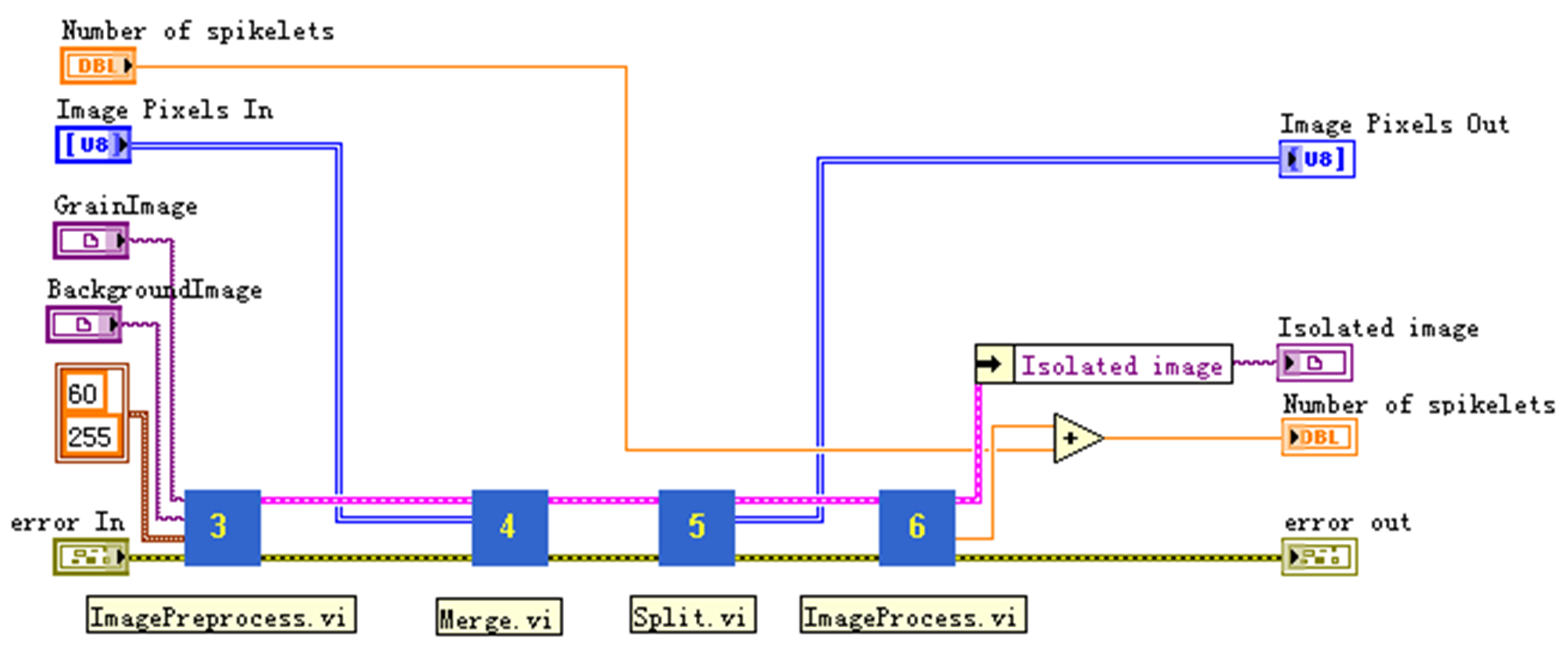

Supplement: Additional file 3 — Source code file 2. ImageAnalysis_TotalSpikeletImage.vi was developed for processing a single total-spikelet image. [file 1746-4811-7-44-S3.TIFF]

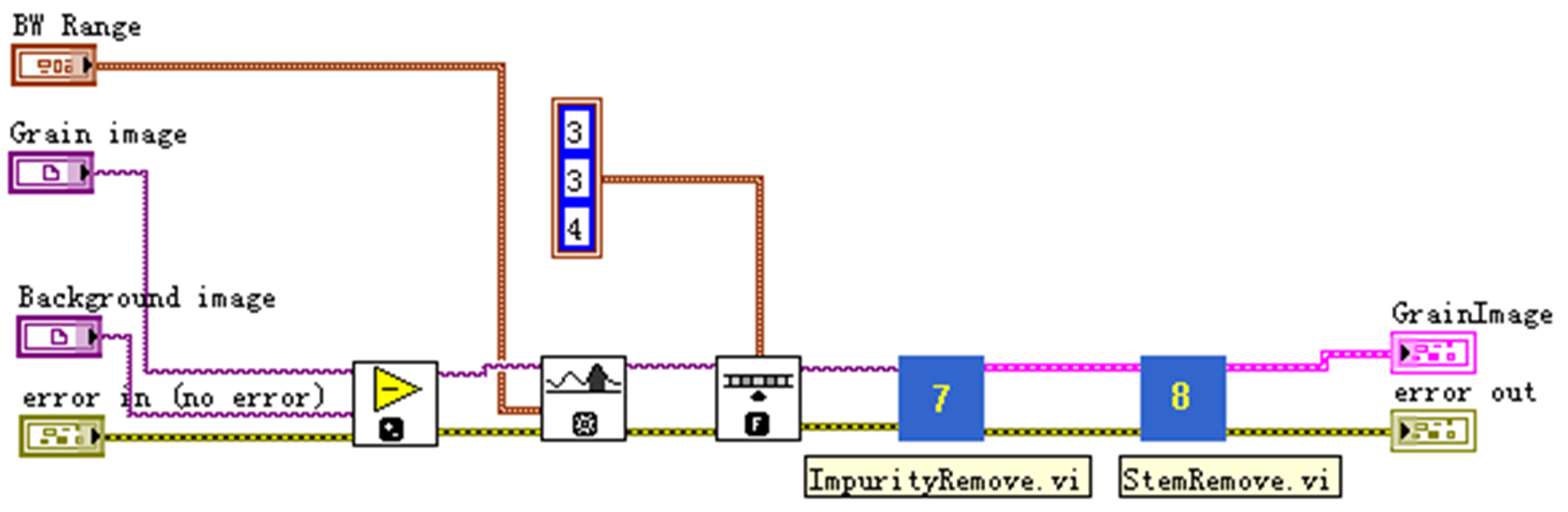

Supplement: Additional file 4 — Source code file 3. ImagePreProcess.vi executed image segmentation and impurity removal. [file 1746-4811-7-44-S4.TIFF]

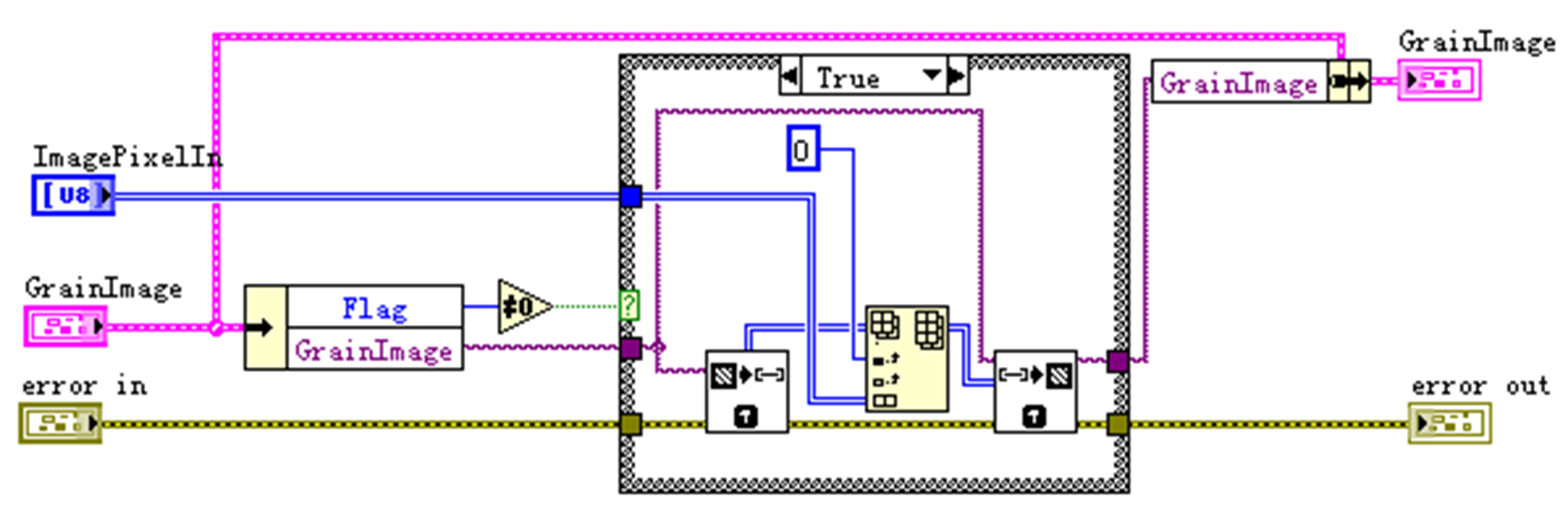

Supplement: Additional file 5 — Source code file 4. In continuous image acquisition, some grains may exist both in the bottom border of the previous image and in the top border of the subsequent image. Merge.vi. merged the object at the bottom border of the previous image with the other part of the object in the subsequent image. [file 1746-4811-7-44-S5.TIFF]

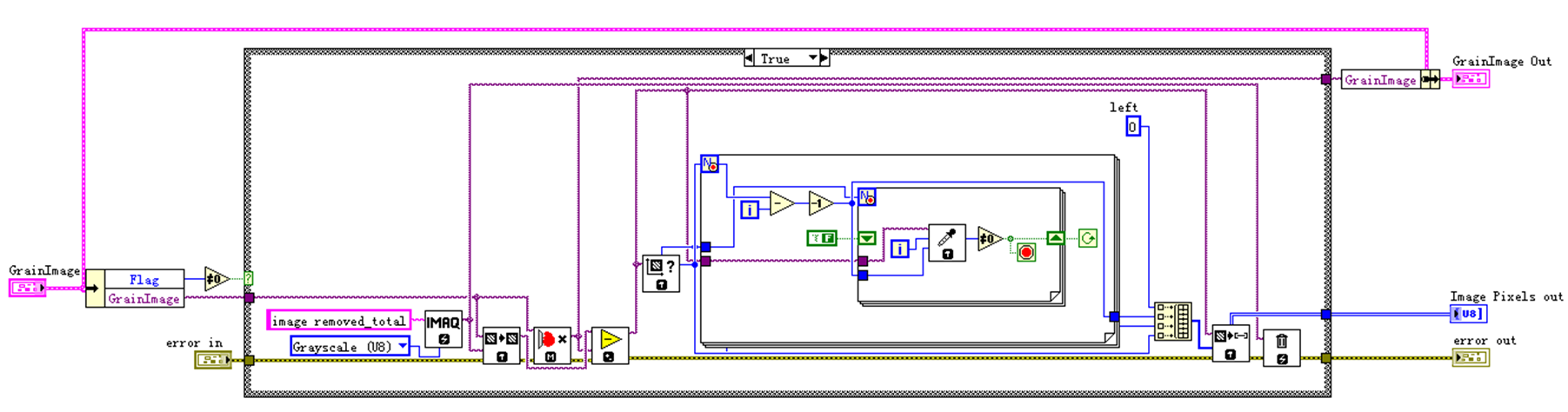

Supplement: Additional file 6 — Source code file 5. Split.vi extracted objects at the bottom border in the current image. [file 1746-4811-7-44-S6.TIFF]

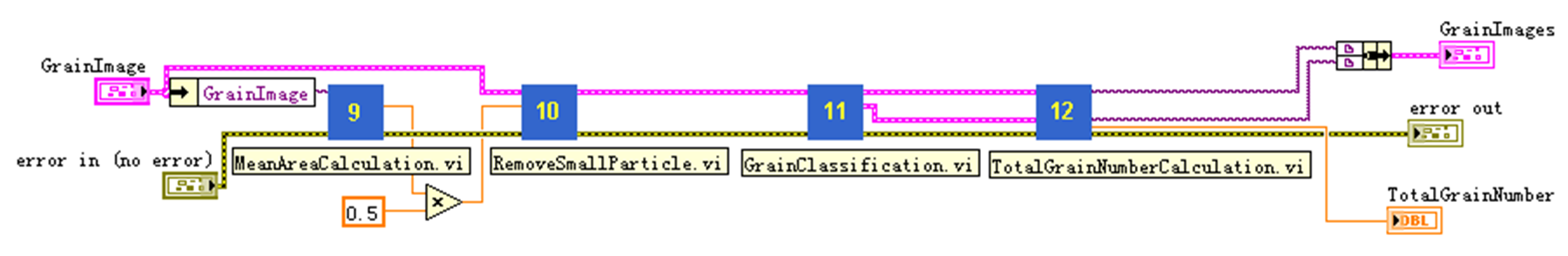

Supplement: Additional file 7 — Source code file 6. ImageProcess.vi removed small particles and calculated spikelet number in an image. [file 1746-4811-7-44-S7.TIFF]

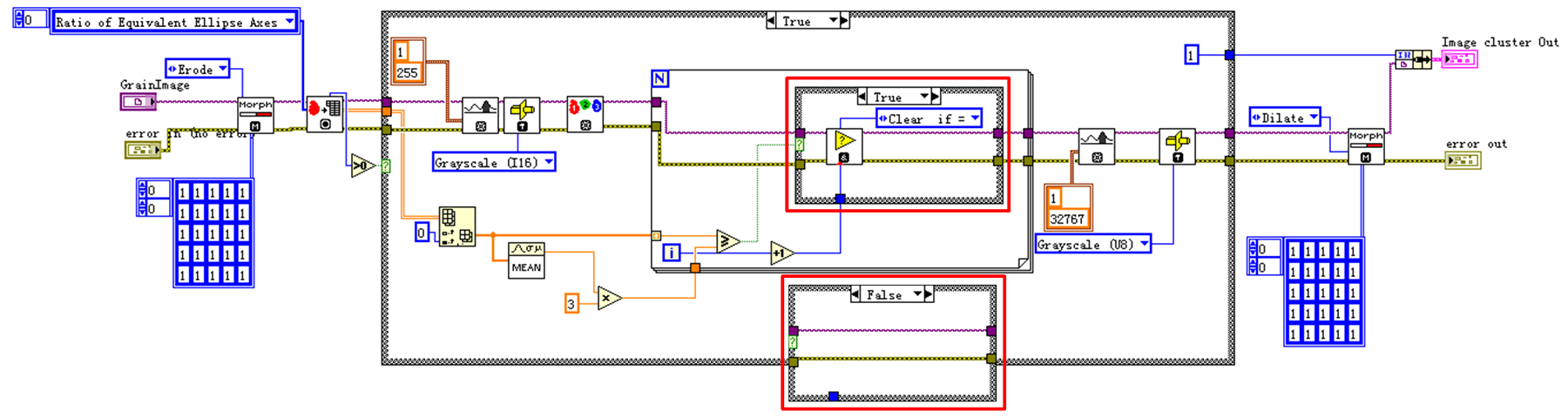

Supplement: Additional file 8 — Source code file 7. ImpurityRemove.vi removed objects with a length-width ratio greater than three times that of the spikelets. [file 1746-4811-7-44-S8.TIFF]

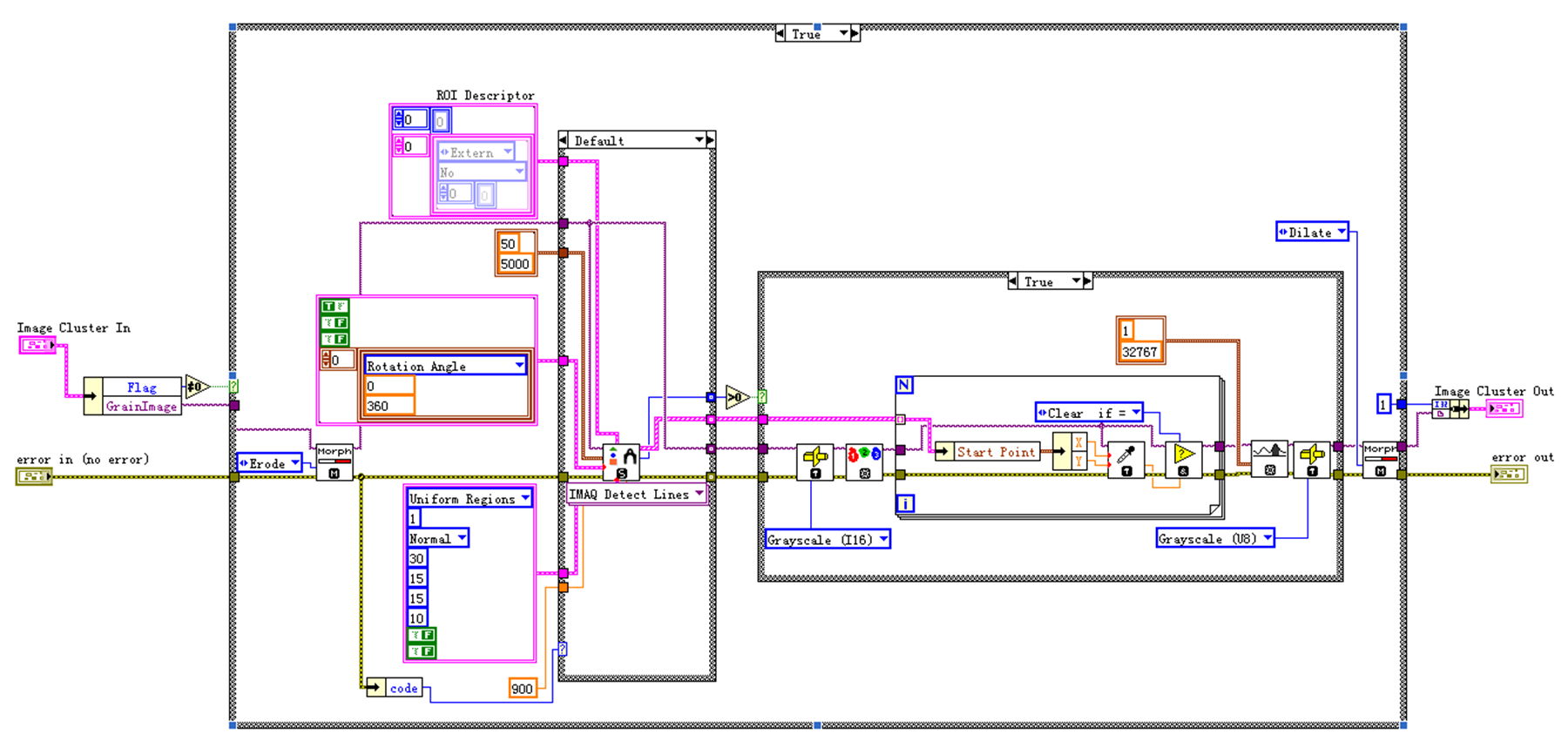

Supplement: Additional file 9 — Source code file 8. StemRemove.vi removed branches with length-width ratios less than three times that of the spikelet using the "IMAQ detect line" operation. [file 1746-4811-7-44-S9.TIFF]

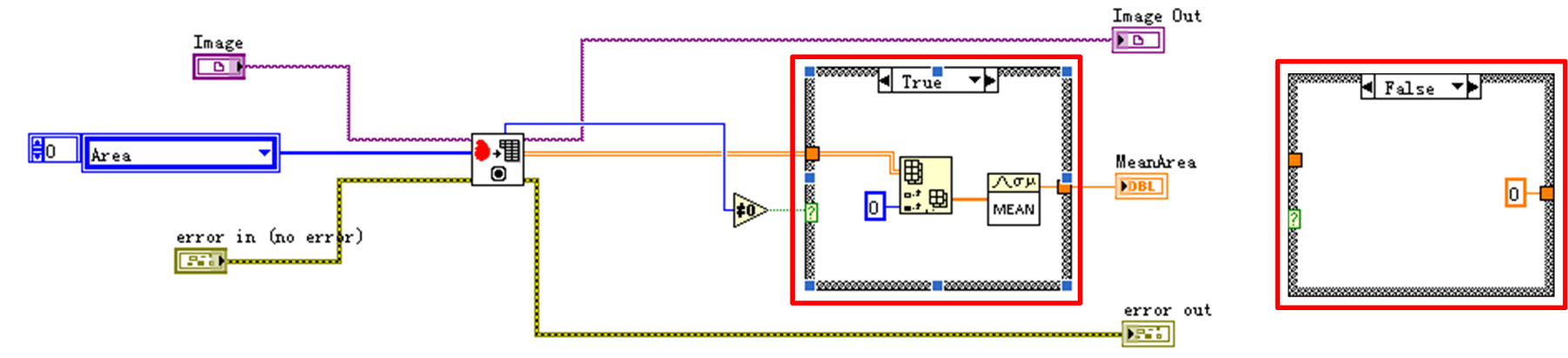

Supplement: Additional file 10 — Source code file 9. MeanAreaCalculation.vi calculated the average area of the spikelets. [file 1746-4811-7-44-S10.TIFF]

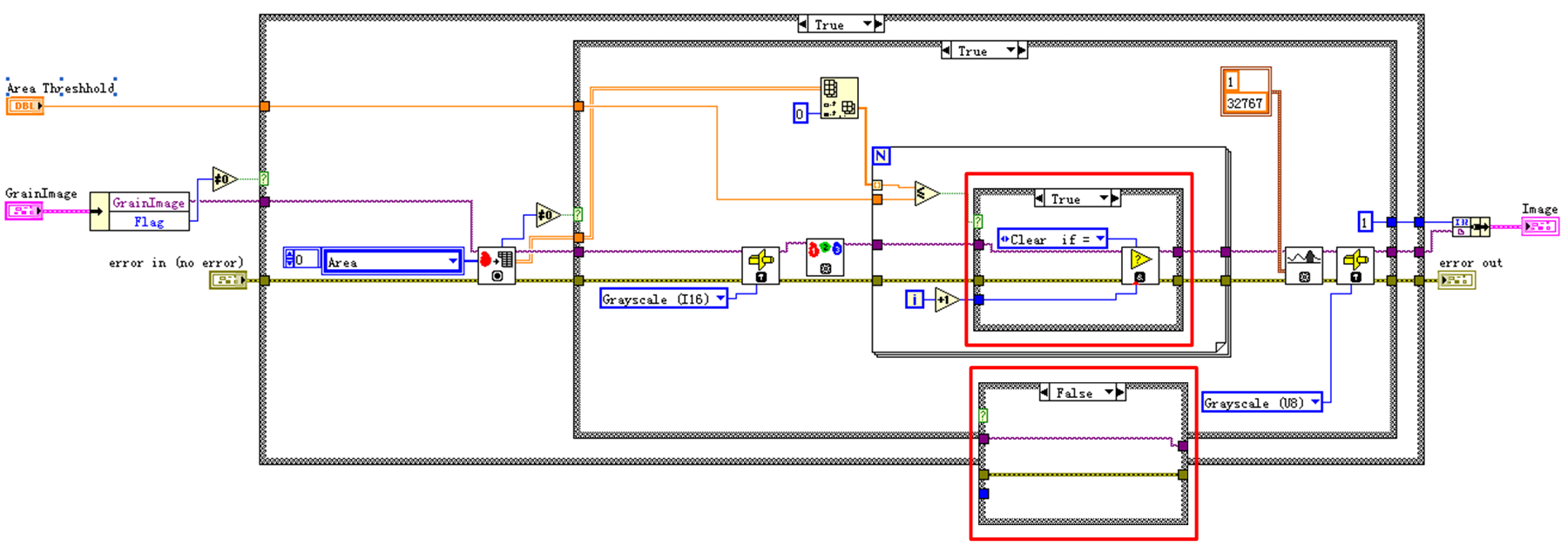

Supplement: Additional file 11 — Source code file 10. RemoveSmallParticle.vi removed small regions with an area less than the defined area threshold. [file 1746-4811-7-44-S11.TIFF]

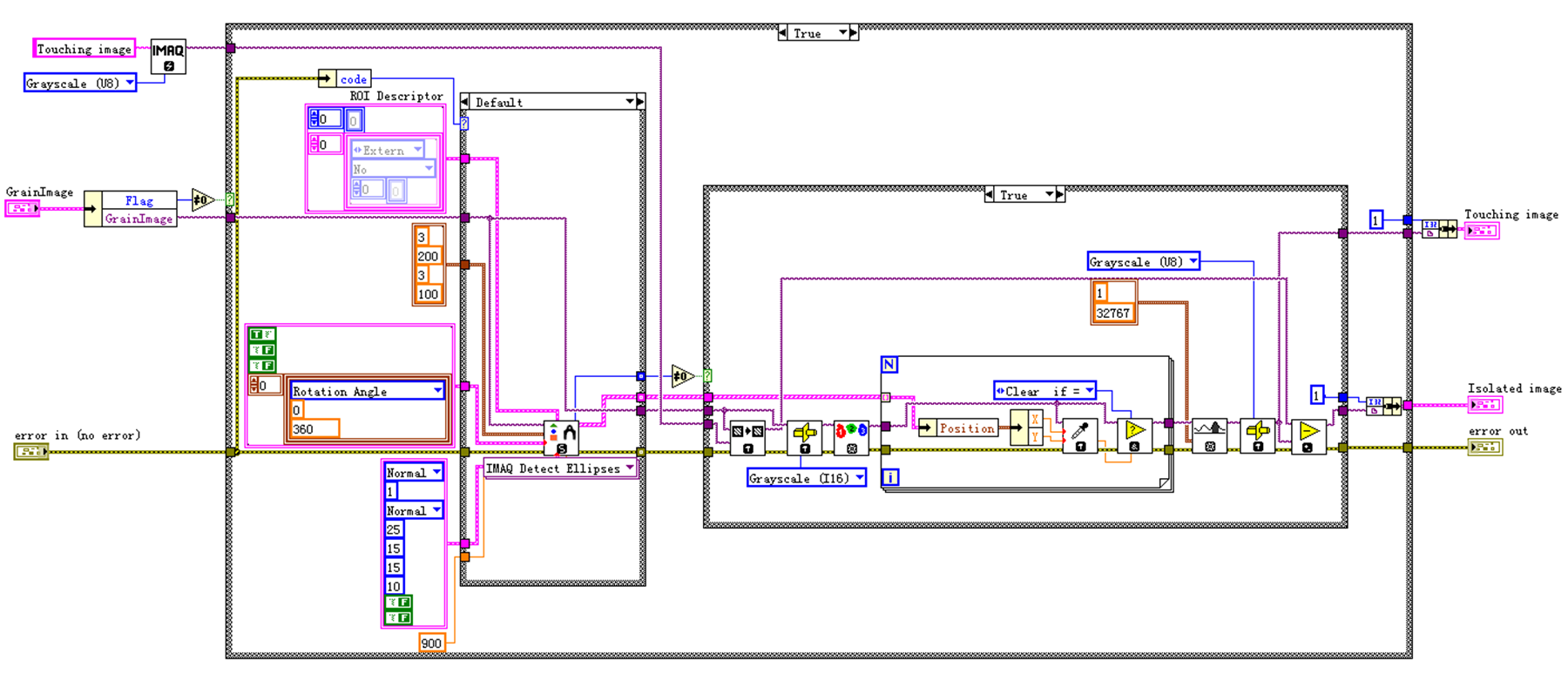

Supplement: Additional file 12 — Source code file 11. GrainClassification.vi divided the original image into two images: one image with only isolated spikelets (isolated image) and the other with only touching spikelets (touching image). [file 1746-4811-7-44-S12.TIFF]

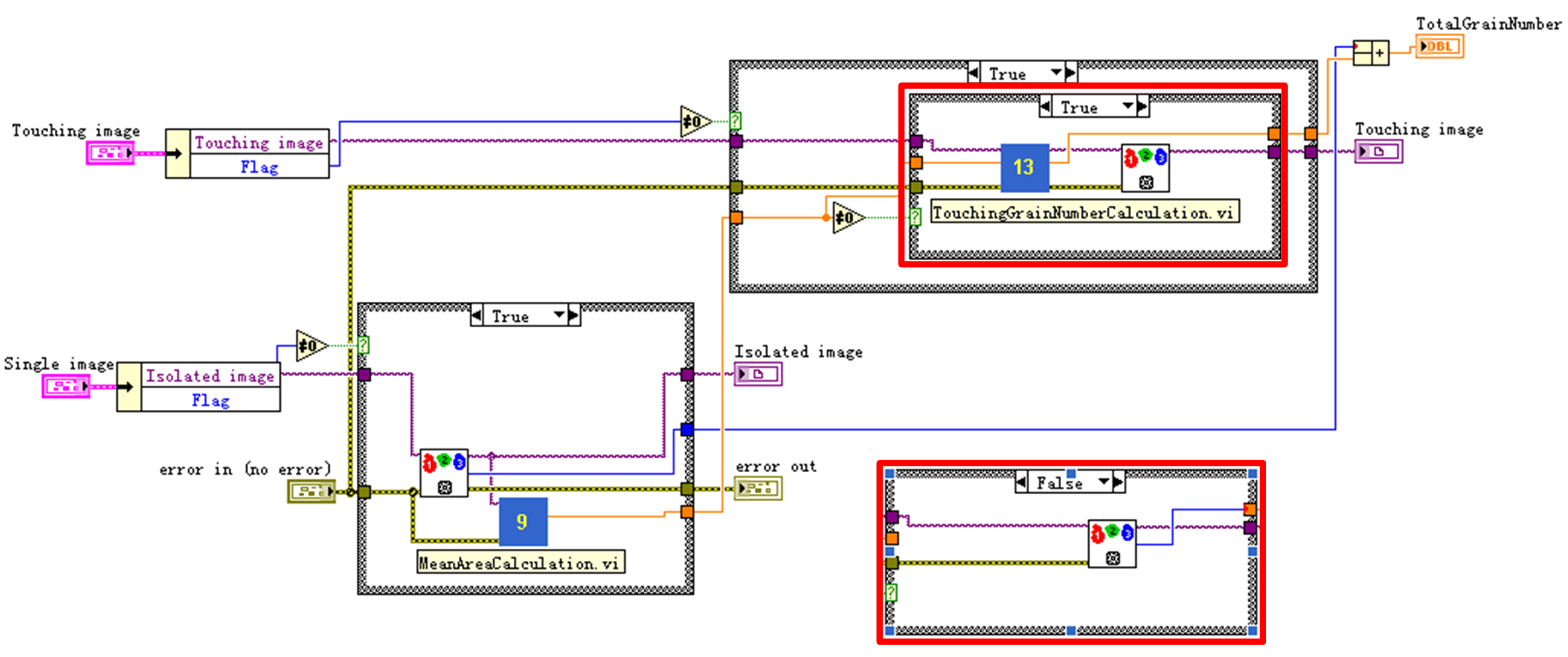

Supplement: Additional file 13 — Source code file 12. TotalGrainNumberCalculation.vi determined the spikelet number in the original image by summing up the spikelet number in the isolated image and the spikelet number in the touching image. [file 1746-4811-7-44-S13.TIFF]

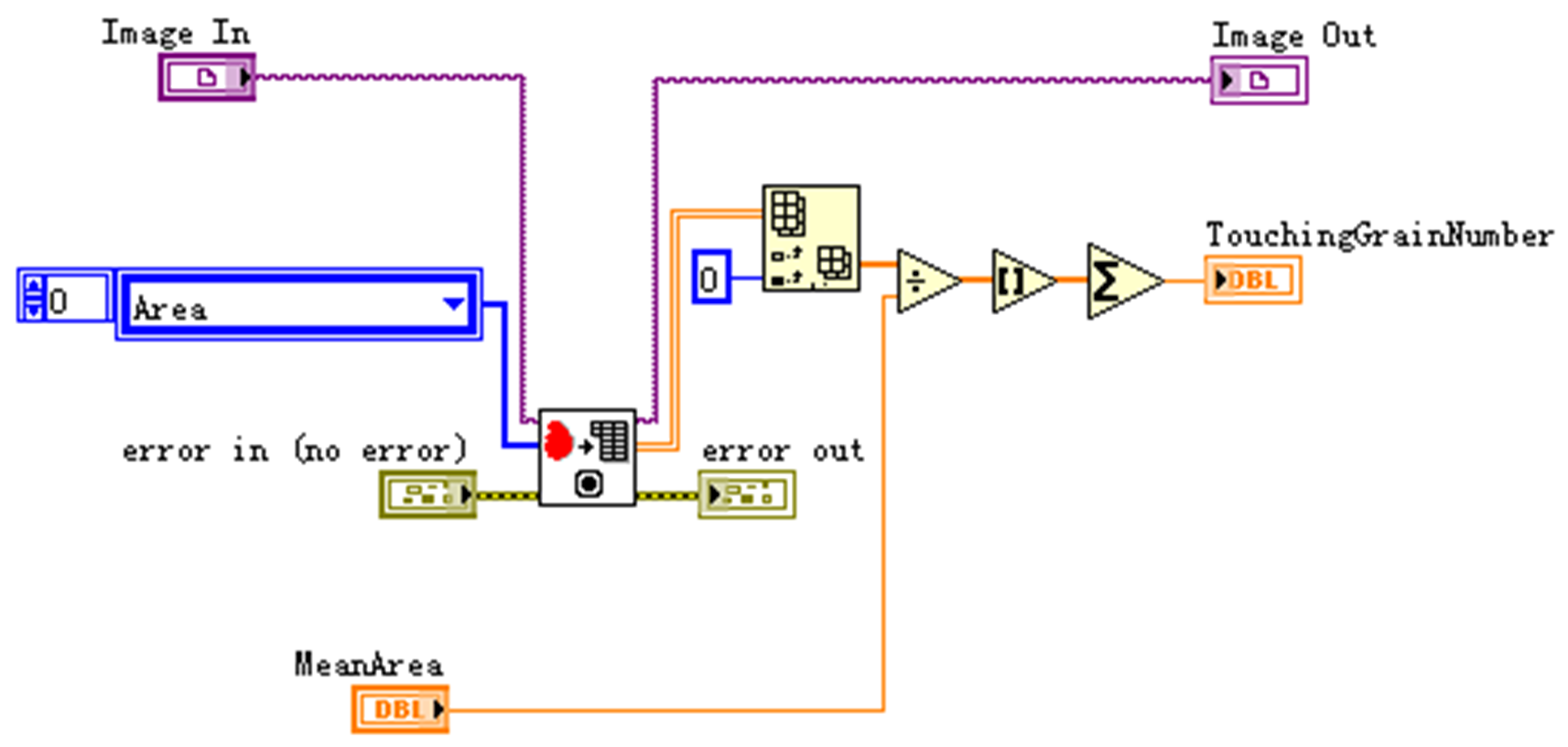

Supplement: Additional file 14 — Source code file 13. TouchingGrainNumberCalculation.vi calculated the spikelet number in the touching image. [file 1746-4811-7-44-S14.TIFF]

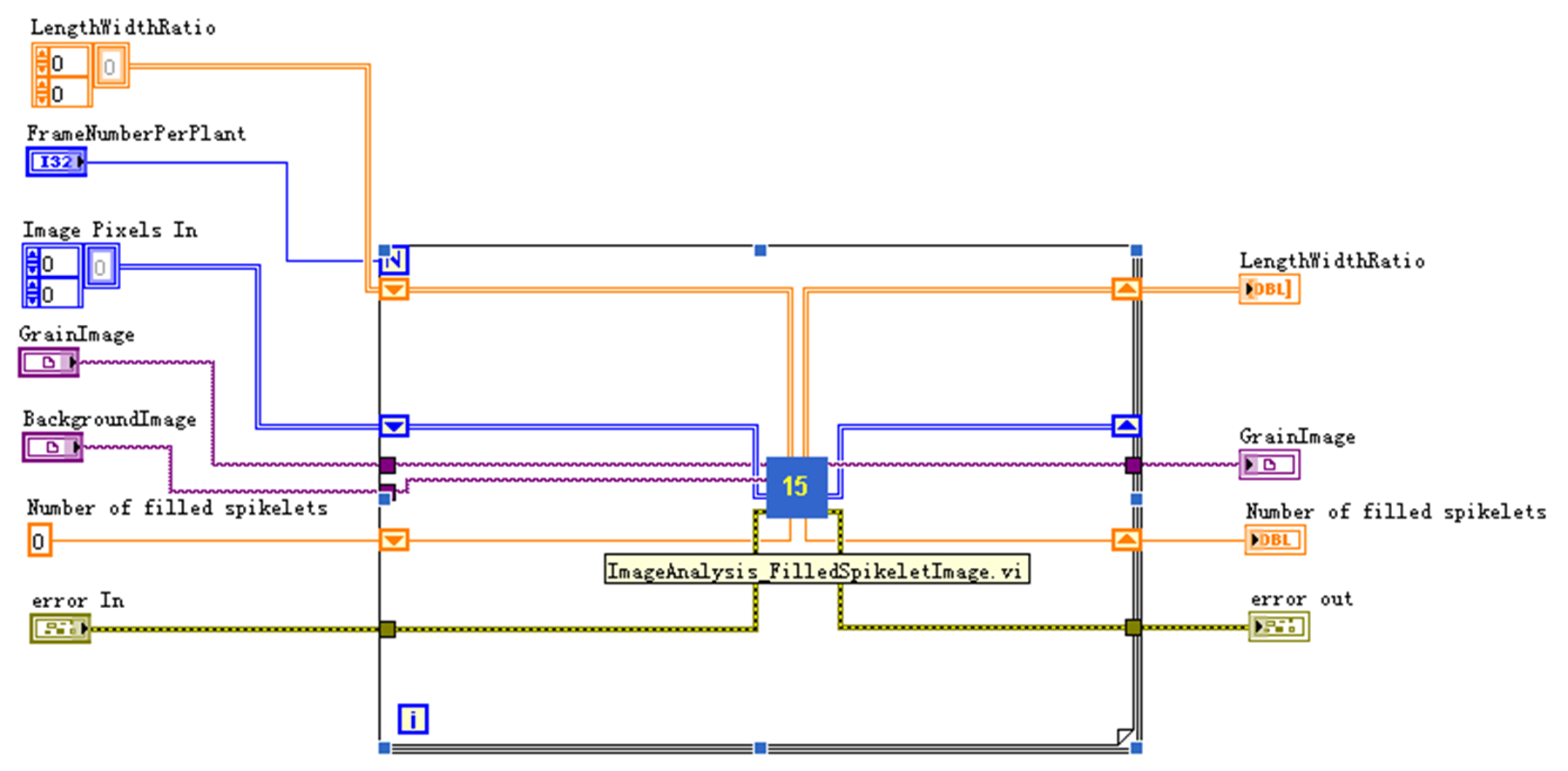

Supplement: Additional file 15 — Source code file 14. ImProcessPerPlant_FilledSpikeletImage.vi was used for processing filled-spikelet images of one plant (20 images in the developed facility). The number of filled spikelets of the evaluated plant was calculated as the sum of the spikelet numbers in all 20 filled-spikelet images. Note that in on-line measurements, ImProcessPerPlant_FilledSpikeletImage.vi functions were included in a 'queue' structure to allow images to be analyzed in the computer simultaneously while the cameras were acquiring new images. [file 1746-4811-7-44-S15.TIFF]

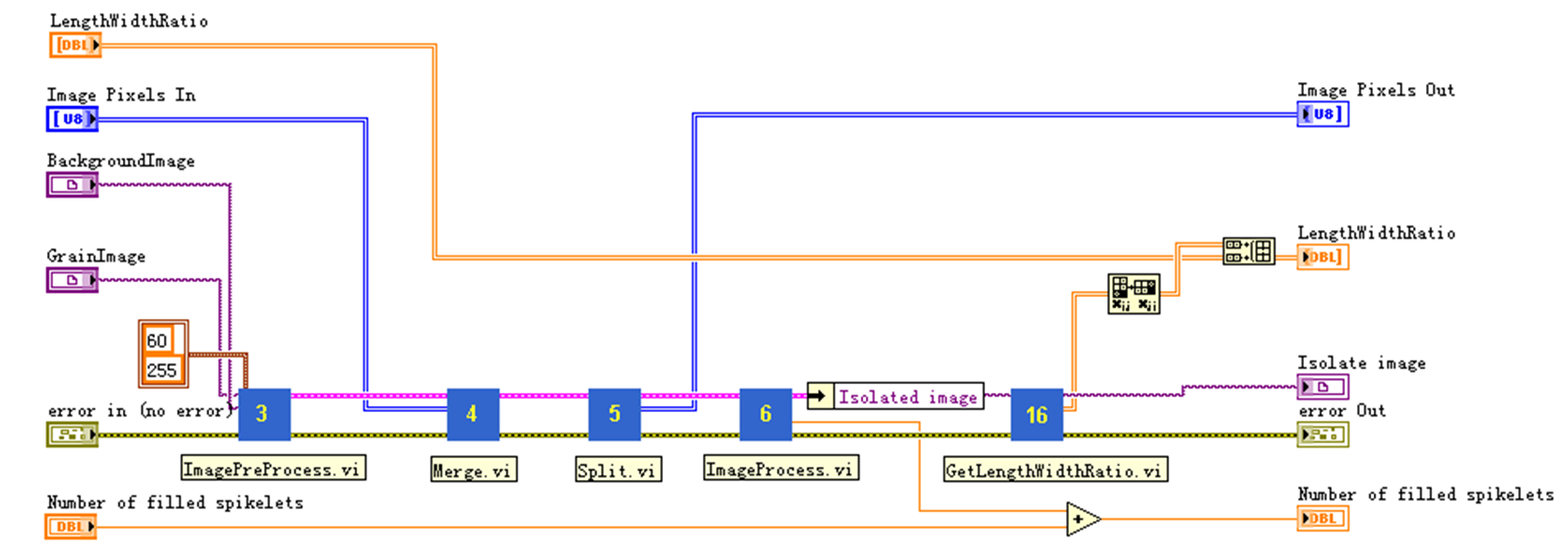

Supplement: Additional file 16 — Source code file 15. ImageAnalysis_FilledSpikeletImage.vi was developed for processing a single filled-spikelet image. [file 1746-4811-7-44-S16.TIFF]

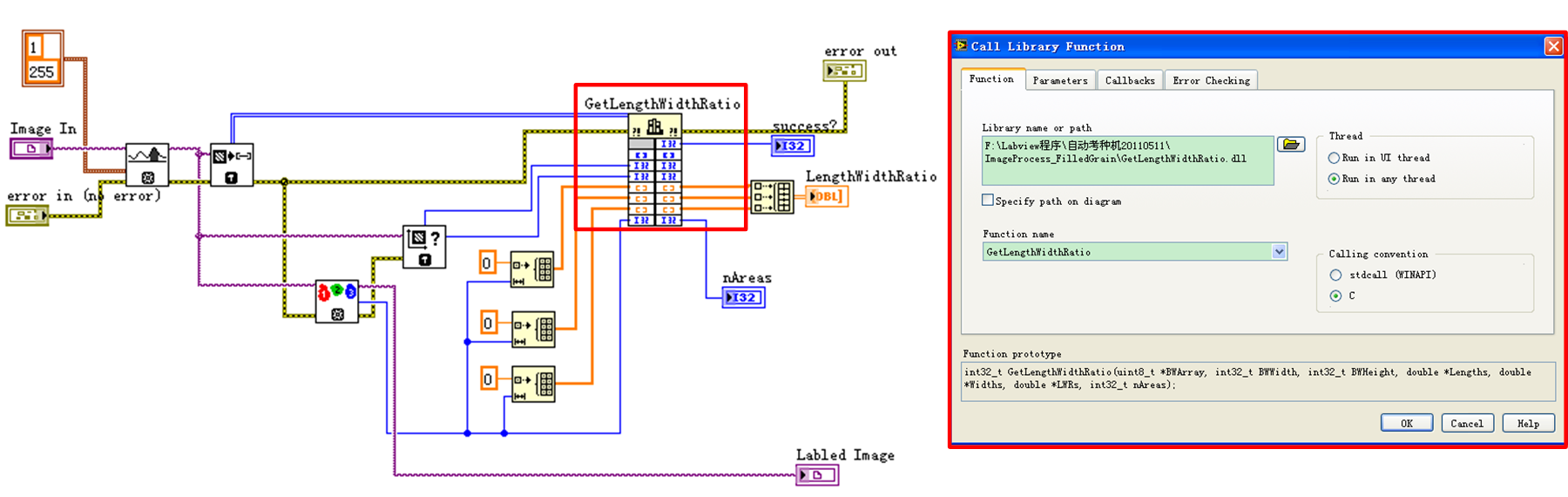

Supplement: Additional file 17 — Source code file 16. GetLengthWidthRatio.vi calculated the length, width, and length-width ratio for each isolated grain. [file 1746-4811-7-44-S17.TIFF]
